# Novel cultivated endophytic *Verrucomicrobia* reveal deep-rooting traits of bacteria to associate with plants

**DOI:** 10.1038/s41598-020-65277-6

**Published:** 2020-05-26

**Authors:** Wiebke Bünger, Xun Jiang, Jana Müller, Thomas Hurek, Barbara Reinhold-Hurek

**Affiliations:** 1 Department of Microbe-Plant Interactions, University of Bremen, Bremen, Germany; 2 Present Address: Department of Botany, University of Bremen, Bremen, Germany

**Keywords:** Soil microbiology, Bacteria, Symbiosis

## Abstract

Despite the relevance of complex root microbial communities for plant health, growth and productivity, the molecular basis of these plant-microbe interactions is not well understood. *Verrucomicrobia* are cosmopolitans in the rhizosphere, nevertheless their adaptations and functions are enigmatic since the proportion of cultured members is low. Here we report four cultivated *Verrucomicrobia* isolated from rice, putatively representing four novel species, and a novel subdivision. The aerobic strains were isolated from roots or rhizomes of *Oryza sativa* and *O. longistaminata*. Two of them are the first cultivated endophytes of *Verrucomicrobia*, as validated by confocal laser scanning microscopy inside rice roots after re-infection under sterile conditions. This extended known verrucomicrobial niche spaces. Two strains were promoting root growth of rice. Discovery of root compartment-specific *Verrucomicrobia* permitted an across-phylum comparison of the genomic conformance to life in soil, rhizoplane or inside roots. Genome-wide protein domain comparison with niche-specific reference bacteria from distant phyla revealed signature protein domains which differentiated lifestyles in these microhabitats. Our study enabled us to shed light into the dark microbial matter of root *Verrucomicrobia*, to define genetic drivers for niche adaptation of bacteria to plant roots, and provides cultured strains for revealing causal relationships in plant-microbe interactions by reductionist approaches.

## Introduction

In land plants, roots are the primary site for interactions with diverse microbes, which are recruited from soil and filtered by the plant^1^. Plants have probably evolved along with their associated microbes for millions of years. The fossil record suggests that bacteria were already endophytic^2^ in plants with simple rhizoid-based rooting systems, during the early evolution of roots 400 million years ago^3^. Current results of reverse genetics and community structure illustrate that endophytic colonization is an active process requiring specific traits of bacteria to establish inside^1,4^. It is known for eukaryotes^5^ and prokaryotes^6^ that exposure of different populations to similar environments may result in similar features because of parallel selection in their genomes. Thus, it is conceivable that bacteria of different taxonomic background may share characteristics for a plant-associated or endophytic lifestyle.

However, how bacteria adapt to and interact with plants is a fundamental question for understanding the molecular basis of root microbiome functions. To discover causal relationships of underlying principles, reductionist studies use single isolates or reconstitution experiments from synthetic communities (large pure culture collections isolated from plants) as promising tools^7^. Yet, in the domain Bacteria many phyla have no or few cultivated representatives^8^, with more than 88% of all isolates belonging to only four phyla^9^. Also in the highly diverse phylum *Verrucomicrobia*^10^, the view on bacterial capacities is skewed because cultured strains are strongly underrepresented. The generally poor cultivation success of *Verrucomicrobia* has impaired progress in understanding their ecology and capacities^11^.Culture-independent methods suggest verrucomicrobial communities in many diverse biomes including soils^11^, where they drive biogeographical pattern changes^12^. Even in these studies, primer bias in commonly used PCR primers may lead to an underestimation of 16S rRNA genes during amplification^13^. With improved primer sets, a high relative abundance of *Verrucomicrobia* was found in grassland soils (35%) and even 52% in subsoils of a coniferous forest^13^. *Verrucomicrobia* are also cosmopolitans in the rhizosphere: in addition to *Arabidopsis thaliana*^14^, poplar^15^, and maize^16,17^ rhizosphere soils, rice roots^18,19^ harbour a considerable verrucomicrobial population (4–6%)^20^, and *Verrucomicrobia* were recently reported to constitute even 25% of the endorhizosphere population^21^. Due to the lack of isolates, only few studies focused on the possible ecological importance or niche adaptation of *Verrucomicrobia* in microbe-plant interactions^22,23^, and no endophytic strains are available. Concomitantly, in published synthetic communities of plant-associated microbes *Verrucomicrobia* are lacking, for example for *Arabidopsis*^24^ or poplar and maize^25^. Therefore, we attempted to isolate root-associated *Verrucomicrobia* in order to evaluate their root colonization capacity, plant promotion characteristics, and putative genomic adaptations to root-associated lifestyle in this phylum, as it is distant to common root endophytes.

Here we report the isolation of four novel, divergent strains of *Verrucomicrobia* from rice roots or rhizomes and thus extend the cultivated root microbiota to this phylum. Two strains were demonstrated for the first time to be root endophytic in gnotobiotic cultures, a novel feature of *Verrucomicrobia*. Characteristics that are common to other plant growth-promoting bacteria such as *Proteobacteria*, were surprisingly also detected in the isolates of the distant phylum *Verrucomicrobia*, which suggests widespread similar genetic drivers of niche adaption and functional diversification. Across-phylum comparison of protein domains encoded in genomes of well-studied reference endophytes, root surface and soil bacteria, allowed to identify novel signature protein domains for compartments, and thus provided a broader insight in adaptations for plant-associated lifestyle that are common in distant phyla.

## Results and Discussion

### Isolation of novel *Verrucomicrobia* from rice, *Oryza* spp

**Figure 1:**
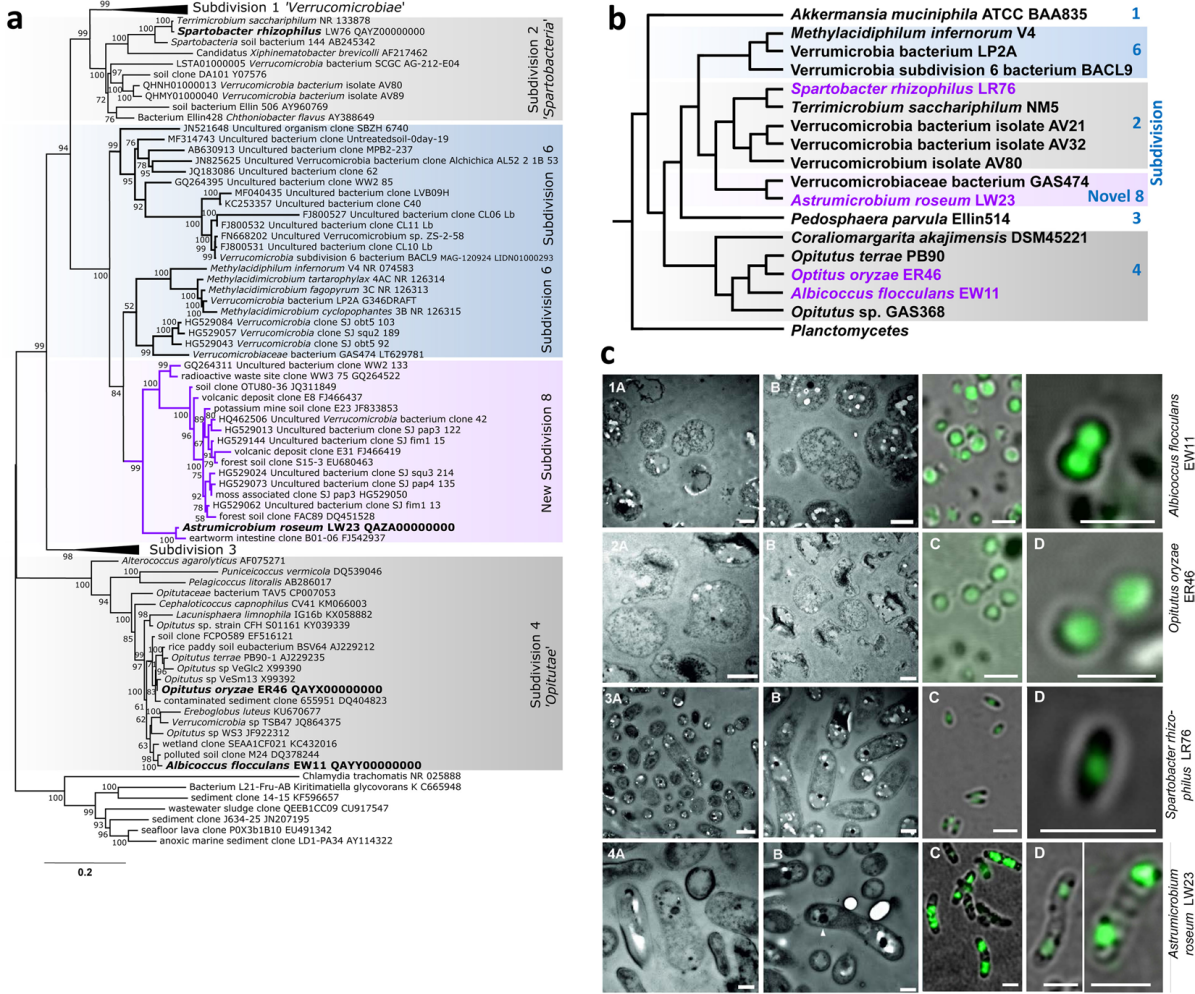
Molecular phylogenetic (**a**), phylogenomic (**b**) and cell morphology (**c**) analysis of the novel *Verrucomicrobia* isolates. **(a**) Molecular phylogeny based on based 16S rRNA gene sequences. Novel isolates of *Verrucomicrobia* in bold, putative novel subdivision 8 in purple. Almost complete 16S rRNA gene sequences were aligned with reference sequences from representatives of every subdivision of the phylum *Verrucomicrobia*. The evolutionary history was inferred by using the Maximum Likelihood method implemented in IQ-TREE. The percentage of trees in which the associated taxa clustered together is shown next to the branches. The tree is drawn to scale, with branch lengths measured in the number of substitutions per site. There were a total of 1228 positions in the final dataset, and *Chlamydia trachomatis (NR 025888)* was used as outgroup. **(b)** Phylogenomic relationships of *Verrucomicrobia* inferred by the whole-genome-based CVTree approach. Genomes or metagenomes covering all known subdivisions were selected, and novel isolates (purple) included. Analysis by CVTree3 with K = 6. **(c)** Cell morphology of strains EW11(1), ER46(2), LR76(3), and LW23(4). (A, B) Transmission electron micrographs of thin sections. (C, D) Images of cells stained with SYBR green to reveal condensed nucleic acids inside cells by Confocal Laser Scanning Microscopy. White arrowhead, electron-dense material. Scale bars indicate 500 nm (1–2 A + B, 3–4 B), 1000 nm (3–4 A) or 2 µm (C,D). Endospores were not observed. Specimen preparation through conventional fixation and dehydration lead to an irregular, scattered shape of some cells (2B) as described for other *Verrucomicrobia*^88^.

In order to obtain cultivable *Verrucomicrobia*, we subjected roots of *O. sativa* and roots and rhizomes of an undomesticated species of rice, *O. longistaminata*, to a large scale cultivation approach. Different compartments were targeted - surface and endosphere after effective surface sterilization. Among a larger culture collection of roughly 250 strains of mainly *Proteobacteria, Actinobacteria* and *Firmicutes* (not shown), four divergent *Verrucomicrobia* were detected that had been isolated under aerobic culture conditions: Small colonies of slow-growing bacteria were found among fast-growing strains upon extended incubation of agar plates. Strains LW23, ER46 and LR76 were obtained from the rhizoplane, rhizome endosphere, and rhizome surface of *O. longistaminata*, respectively, whereas strain EW11 was isolated from the root endosphere of *O. sativa*. Phylogenetic analysis of almost complete 16S rRNA gene sequences placed strain LR76 in subdivision 2, and EW11 and ER46 in subdivision 4 (Fig. 1a), out of seven known subdivisions proposed for *Verrucomicrobia*^10,26^. Strain LW23 clustered as sister lineage to methylotrophs of subdivision 6, without cultured bacteria being closely related (Fig. 1a). Phylogenetic, phylogenomic (Fig. 1a,b) and physiological features suggest that strain LW23 belongs to a novel subdivision 8 (Supplementary Note 1, Supplementary Tables S1,S2). The putative new subdivision consists entirely of uncultured bacteria from diverse ecological habitats, with exception of strain LW23 and *Verrucomicrobiaceae* bacterium GAS474 from forest soil: for example intestine of the earthworm *Eisenia fetida*^27^ (97% identity), a low-level radioactive waste site^28^ (91% identity), marine (coral) and aquatic moss, freshwater lake, as well as terrestrial habitats. Therefore, it is difficult to predict which physiological requirements are needed for isolation of more cultured strains belonging to this ecologically diverse new subdivision. Previous subdivision numbers 5 and 7 were meanwhile assigned to the rank of distinct phyla closely related to *Planctomycetes*, *Verrucomicrobia* and *Chlamydiae*, namely ‘*Lentisphaerae’*^29,30^ and ‘*Kiritimatiellaeota’*^31^; therefore these subdivision numbers were not re-used by us for the novel subdivision.

Cultivated root-colonizing rhizosphere soil isolates were described only within *Verrucomicrobia* subdivision 1 up to now^23,32^. In subdivision 4, “*Opitutae*” were detected in the rhizosphere soil of pine by culture-independent methods^33^, but we report the first cultivated root-associated members: strain ER46, related to a paddy soil isolate *Opitutus* sp. VeSm13^34^, and strain EW11, more distantly related to *Verrucomicrobia* sp. TSB47 from termite gut. *Spartobacteria* (subdivision 2) were most commonly found in terrestrial environments^13^, including in rhizosphere soil^33^. The strictly anaerobic *Terrimicrobium sacchariphilum* from rice paddy soil^35^ was the closest cultured relative to strain LW76. Thus our study extended the range of cultivated root inhabitants to three other subdivisions, suggesting a more widespread close association with roots among *Verrucomicrobia*.

A 16S rRNA gene sequence identity below 98.7–99% has been defined as threshold value for species delineation^36^. Above this value, the analysis of the average nucleotide identity (ANI)^37,38^ between genomes is used to compare the genetic relatedness among putative species. ANI values of ~94% correspond to the traditional 70% DNA-DNA hybridization boundary differentiating species from each other^39^. Comparison of genomes of the novel strains to those of known related species yielded less than 89% (Supplementary Table S3), confirming the rank of at least novel species. As 16S rRNA gene identities less than 94.5% suggest distinct genera^40^, all strains except ER46 probably constitute novel genera (Supplementary Table S3). Formal description of the new taxa will be submitted elsewhere, as names *Astrumicrobium roseum* LW23, *Opitutus oryzae* ER46, *Spartobacter rhizophilus* LR76, and *Albicoccus flocculans* EW11 are proposed.

### Cell compartmentalization in *Verrucomicrobia* isolates extends to the novel subdivision

Morphological analysis suggested that cell compartmentalization in *Verrucomicrobia* extends to the novel subdivision. Some members of the *Verrucomicrobia* possess a compartmentalized cell plan analogous to that found in phylum *Planctomycetes*^31,41^, detected in cells of subdivision 1, 2 and 3^41^. SYBR green staining of nucleic acids showed clearly distinct small zones of fluorescence in confocal laser scanning microscopy (CLSM) analysis of *Astrumicrobium roseum* LW23 (Fig. 1c), indicating that nucleoid-containing cell compartments also occur in the novel proposed subdivision 8. In *Spartobacter rhizophilus* LR76 from subdivision 2, compartmentalized staining was observed as well, but it was not evident in cocci EW11 and ER46 from subdivision 4 (Fig. 1c).

### Endophytic colonization pattern of novel *Verrucomicrobia*

**Figure 2:**
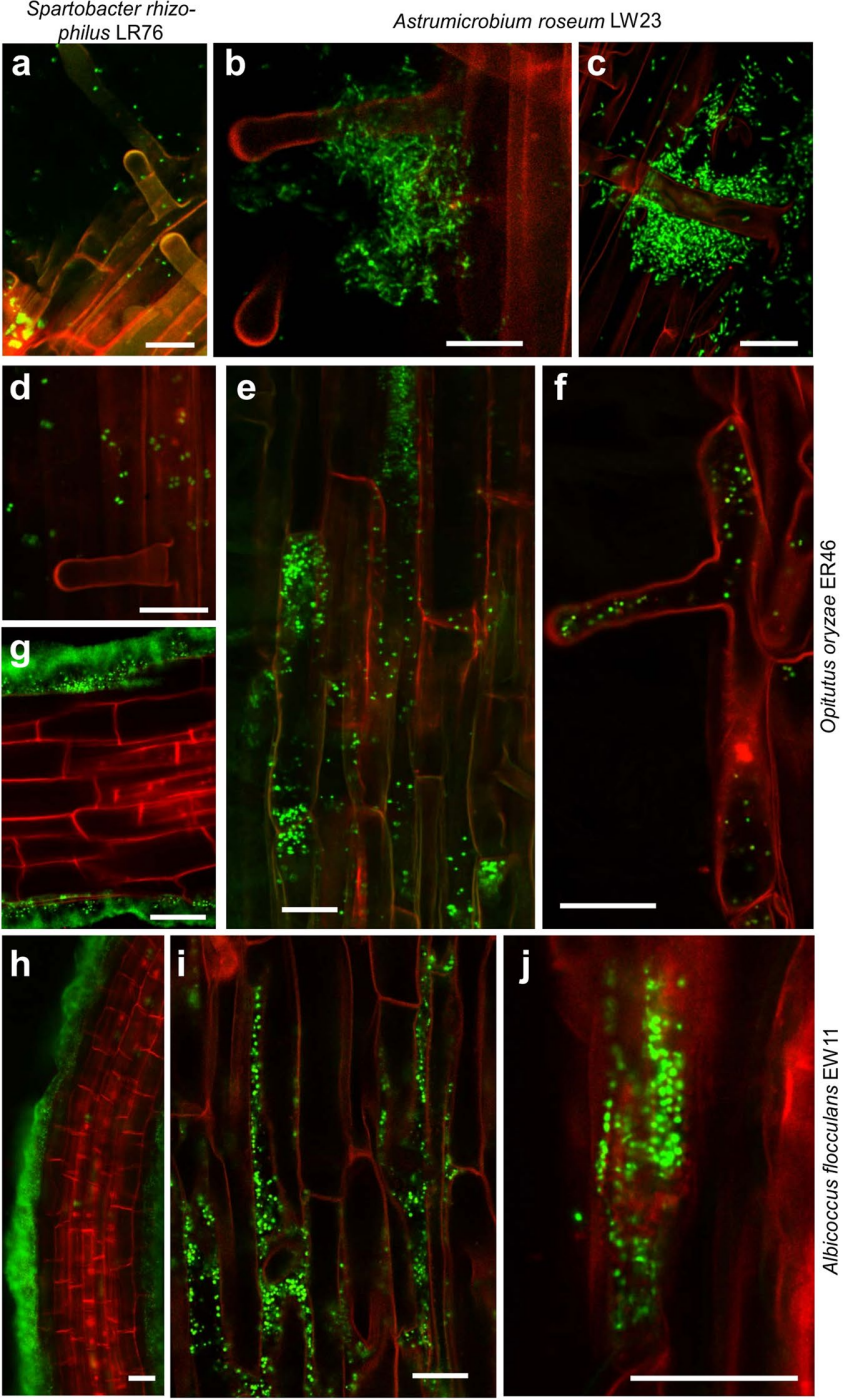
Confocal Laser Scanning Microscopy of rice roots at 8 days post inoculation in hydroponic gnotobiotic culture. Rice variety *O. sativa* ssp. *japonica* cv. Nipponbare, inoculated under gnotobiotic conditions and incubated for 7d. Roots were stained with SYBR Green to visualize bacteria. (**a**) Colonization of strain LR76, of strain LW23 (**b,c**), of strain EW11 (**e,f**), and of strain ER46 (**d,g–j**). Bars indicate 100μm (**a**), 50 μm (**b–e,g,h**) or 20 μm (**f**), respectively. Streaking of plant medium on RSA agar did not reveal any contamination. The non-inoculated control pants showed microscopically no bacterial colonization (Supplementary Fig. 1), and generally in this seed batch, intrinsic endophytes were not detected in any experiment.

Root-associated bacteria may occupy different niches - the rhizosphere soil, root surface (rhizoplane), or endorhizosphere - and thus show increasing intimacy of the interaction with plants as well as genomic adaptations to their niche^1^. Microscopic validation is most important for the assessment of a truly endophytic lifestyle^1,42^. Therefore, we tested the isolate’s colonization capacities for rice in a hydroponic cultivation system under gnotobiotic conditions. Differential colonization patterns were detected for the novel *Verrucomicrobia* by fluorescence microscopy (Supplementary Fig. 1) and CLSM (Fig. 2); microbial contaminations were not detected in any of the uninoculated controls (Supplementary Fig. 1). *S. rhizophilus* LR76 colonized the root surface, while *A. roseum* LW23 attached to roots and root hairs in large aggregates. For none of both, endophytic colonization was observed. Only isolates originating from the endosphere were proven to be endophytes: *O. oryzae* ER46 attached only weakly to the root surface. Nonetheless, bacteria colonized roots endophytically, clearly visible inside rhizodermis cells and root hairs (Fig. 2e,f; Supplementary Videos 1, 2). In contrast, *A. flocculans* EW11 colonized the root surface very evenly, densely, and in some cases covered roots in large biofilms (Fig. 2g,h). Endophytic colonization was detected in intercellular spaces and inside root cells (Fig. 2i,j and Supplementary Videos 3,4). This is the first microscopic proof of endophytic colonization by verrucomicrobia and clearly extends the niche of *Verrucomicrobia* to the root endophytic compartment.

### Protein domains significantly enriched in root-associated bacteria

Validation of verrucomicrobia strains as endophytes or root surface colonizers permits to elucidate features which are characteristic for a plant-associated or endophytic lifestyle, if they are shared among root-associated but not soil bacteria across diverse phyla. Thus, draft genome sequences were generated for our strains (Supplementary Table S1). For comparison, we chose genomes and metagenomes of distantly related bacteria with well-documented typical lifestyles in endorhizosphere or rhizoplane of various plants, or in soil (Supplementary Table S4). Particularly endophytic lifestyle must be unambiguously demonstrated by microscopic means^42,43^, as commonly used techniques to remove root surface bacteria in culture-independent studies^44^ often fail^1^, and thus the true endomicrobiome picture will be blurred. Rhizoplane bacteria were included, since previous studies for example on *Spartina*^45^ or Kallar grass^46^ had indicated that rhizoplane and endorhizosphere populations may be different from each other; thus they might be differentiated by specific bacterial traits of endophytes that are required for ingress and establishment inside the host^1^. Tested bacteria comprised of diverse phyla, *Verrucomicrobia, Proteobacteria, Acidobacteria*, and *Gemmatimonadetes*.

**Figure 3:**
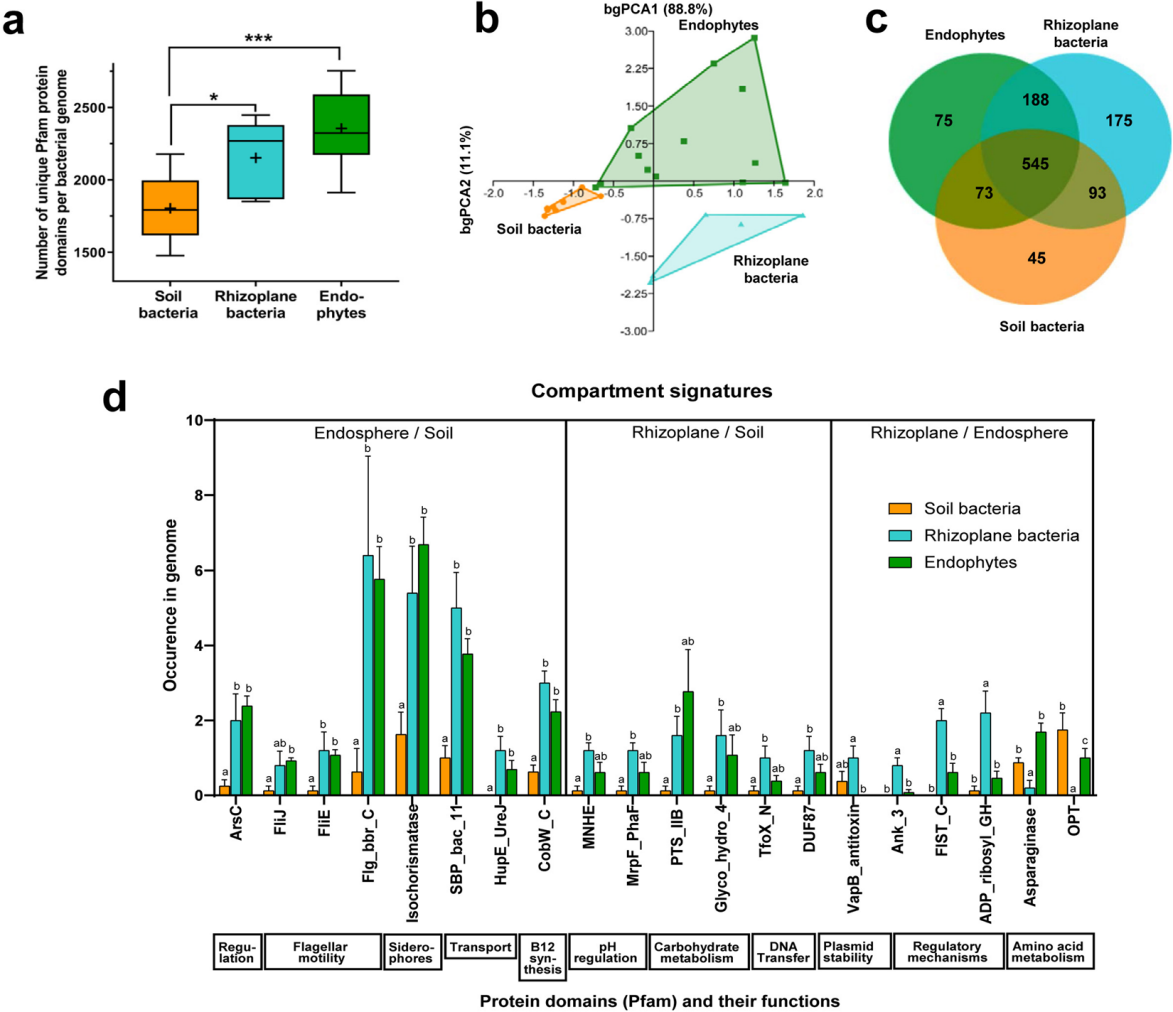
Protein domains differentiating bacterial lifestyles in endophere, rhizoplane and soil across bacterial phyla. Protein domains in (meta)genomes of well-established model organisms typically colonizing endosphere, rhizoplane or soil were compared. (**a**) Abundance of unique protein domains in (meta)genomes of bacteria from different compartments. Box-and-whisker plots for median (center lines), 25th and 75th percentiles (box edges), extreme data points (whiskers), and mean value (plus). Horizontal lines: statistical significance according to analysis of variance (ANOVA) followed by Holm-Sidak’s multiple comparison test, *P* ≤ 0.05 (*) or *P* ≤ 0.001 (***). **(b)** Between-group principal component analysis (bgPCA) of all Pfam protein domains in (meta)genomes of bacteria from the three compartments (*P* < 0.005 for all 3 pairwise comparisons according to PERMANOVA). **(c)** Venn diagram indicating differences and commonalities in Pfam domains of structural proteins encoded in the (meta)genomes from different compartments. The inner circle indicates the number of core protein families shared between all strains. The number of domains shared by all strains of one compartment are given in the outer circles (based on data from Supplementary Table S5). **(d)** Compartment signatures of protein domains. Examples of Pfam domains are given that are significantly enriched in (meta)genomes from endosphere or rhizoplane bacteria; for a given Pfam domain, bars are labelled by different letters if they are significantly different from each other at *P* < 0.05 using the Holm-Sidak method without assuming equal variance. Functions related to the respective domains are given below. Compartments depicted in the same colour in A-D. Model organisms were for soil: *Verrucomicrobia* bacterium isolates AV21, AV32, AV80, *Acidobacteria* bacterium isolate gp1 AA112, *Gemmatimonadetes* bacterium isolate AG11 [soil metagenomes obtained by whole genome shotgun sequencing (WGS)], *Opitutus terrae* PB90-1, *Opitutus* sp. GAS368, *Azoarcus aromaticum* EbN1 (complete genomes); for rhizoplane: *Verrucomicrobia Astrumicrobium roseum* LW23 and *Spartobacter rhizophilus* LR76, *Azospirillum halopraeferans* Au4, *Azoarcus communis* SWuB3, *Azospirillum brasilense* Sp7 (all WGS), and endophytes: *Opitutaceae Albicoccus flocculans* EW11 and *Opitutus terrae* ER46 (WGS), *Azoarcus olearius* BH72, *Herbaspirillum seropedicae* Z67, *Gluconacetobacter diazotrophicus* PAl 5, *Serratia proteamaculans* 568, *Azospirillum* sp. B510, *Pseudomonas putida* W619, *Pseudomonas stutzeri* A1501, *Klebsiella variicola* 342 (previously: *K. pneumoniae* 342), *Enterobacter* sp. 638, *Methylobacterium populi* BJ001, and *Stenotrophomonas maltophilia* R551-3 (complete genome). Based on data from Supplementary Table 7 where details are given. Graphs done with GraphPad Prism.

Our across-phylum quantification of Pfam-domain diversity per genome revealed significant differences for all three compartments (Fig. 3a). The number of different domains per genome was highest in true endophytes, followed by rhizoplane colonizers, and lowest in soil bacteria. This suggests an increased degree of complexity of protein functions with increased intimacy of plant-microbe interaction, as it has been speculated^1^. Niche specialization was also indicated in Principle Component Analysis, which clearly separated Pfam domains of endophytes, rhizoplane bacteria and soil bacteria (Fig. 3b, Supplementary Table S5), with *P* < 0.018 in pairwise ANOSIM comparisons of the compartments. Despite the higher diversity of phyla among soil bacteria selected in our reference collection, soil-related protein domains clustered well and appeared less diverse (Fig. 3b), suggesting high functional similarities. Functional relatedness of endosphere and rhizoplane microbiomes was indicated by a larger overlap of protein domains with each other (188, Fig. 3c) in comparison to soil microbiomes (73 or 93, respectively).

Therefore, a robust quantitative comparison of protein domain occurrence was carried out for genomes representative for the three compartments. It revealed putative functions differentiating all three the lifestyles from each other (Supplementary Table S4). Several domains are discussed which matched also domain occurrence in our novel *Verrucomicrobia* (Fig. 3d). Presence in phylogenetically very distant root-associated phyla, *Proteobacteria* and *Verrucomicrobia*, are a strong indication of lifestyle-adapted traits. Enriched in endosphere compared to soil microbiomes, the most striking group with 19 domains was related to motility by flagella, across phyla. They were almost absent from the soil microbiome but also abundant in many rhizoplane bacteria, exemplified by cytoplasmic chaperone FliJ, basal rod protein domain Flg_bbr_C, and flagellar hook-basal body complex protein FliE (Fig. 3d). Swarming assays corroborated motility of *Verrucomicrobia* endophytes EW11 and ER46. Motility allows the bacteria to move towards the host guided by root exudates and other gradients, and was proven to convey an advantage for endophytic colonization^47^. Significantly enriched in both, endosphere and rhizoplane over soil, were domains related to transport (SBP_bac_11, HupE_UreJ), transcriptional regulation (ArsC), but also synthesis of cobalamin (CobW_C) and putative siderophores (Isochorismatase). In the legume symbiont *Ensifer meliloti*, cobalamin synthesis was proven to play a critical role for survival in the plant host^48^. Biocontrol against plant pathogens may be mediated though competition for iron^49^, by synthesis, secretion and uptake of iron-chelating siderophores. Endophyte EW11 and epiphyte LW23 showed siderophore production in bioassays (Supplementary Note 2, Supplementary Table S6), and also harboured TonB-dependent siderophore receptor genes (*fiu*). Putative TonB-receptor genes were present in all four verrucomicrobial isolates. These domains may thus be regarded as typical for root-associated bacteria.

Several domains which were significantly enriched in rhizoplane versus soil microbiomes but were rare in endophytes indicate that the root surface is a special microniche: it may be a hotspot for DNA exchange, require pH-regulation to combat acidification by roots, and provide carbohydrates: (a) TfoX_N is found in of transcriptional regulators TfoX that positively regulate genes required for DNA transformation (late competence-specific genes); DUF87, now Her_A, is present in proteins related to bacterial conjugation like TraD (transferosome) or TraC (assembly of F-pilins). (b) Rhizoplane microbiomes harboured also significantly more domains related to Na^+^/H^+^ and K^+^/H^+^ antiporters (PhaG_MnhG_YufB, MNHE, MNHB), and proteins involved in pH regulation (MrpF_PhaF) in comparison to soil microbiomes. (c) Close vicinity to roots and their exuded polysaccharides is likely to promote significant enrichment of domains related to sugar and cellobiose transporters or glycolase hydrolases (PTS_EIIC, PTS_IIB/Glyco_hydro_4C, Glyco_hydro_4).

Quantitative domain analysis was even able to functionally differentiate typical endophytes from rhizoplane colonizers. Endophytes appear to specialize towards certain nitrogenous metabolites, because domains of asparaginase, oligopeptide transporters (OPT) and adenosine deaminase (A_deaminase) were significantly enriched and virtually absent in rhizoplane bacteria. Specific for rhizoplane bacteria were several domains related to regulatory proteins which were virtually absent in the endophyte and soil bacteria set: Ankyrin repeats (Ank_3) mediating protein–protein interactions in signal transduction, are far more common in eukaryotes than in bacteria, supporting the recent finding that plant-associated bacteria share proteins that resemble plant proteins^25^; VapB_antitoxin domains of VapB proteins that control plasmid stabilization, often to avoid loss of virulence genes, are supportive of the assumption of active genetic exchange on the root surface. Enriched over endophytes and almost absent in soil bacteria were ADP-ribosylglycohydrolases (ADP_ribosyl_GH) to remove covalent protein modifications of ADP-ribose moieties to alter protein activities; they were particularly abundant in rhizoplane verrucomicrobia; the FIST C domain (FIST_C) is a novel sensory domain present in signal transduction proteins putatively related to amino acid metabolism.

Our approach using well-defined model bacteria provided new insights in niche-specific signature proteins. In contrast, most domains identified as plant-specific in a previous study were only taxa-specific and not significantly enriched across phyla in stringent analyses^25^, which might be entailed by inclusion of a plethora of strains that are not sufficiently niche-specific.

### Other traits for plant growth promotion and colonization in *Verrucomicrobia*

As these are the first *Verrucomicrobia* isolated from roots or rhizomes, additional features related to plant colonization and interaction were of particular interest. Evidence accumulates that *Verrucomicrobia* play critical roles in environmental carbon cycling and (poly)saccharide degradation, as they can have high coding densities for glycoside hydrolase genes^50,51^. According to our analysis, they add a large set of carbohydrate hydrolysing enzymes to the endomicrobiome. Carbohydrate- active enzymes annotated by the dbCAN2 annotation tool^52^ were abundant in the plant isolates, with endophytes EW11 and ER46 harbouring the highest gene density (7.5% or 7.0%, respectively) in comparison to root surface bacteria LR76 and LW23 (6% or 4.4%, respectively). For 163 or 77 of these enzymes of the endophytes, respectively, signal peptides were predicted, suggesting the bacteria are equipped with a large set of exoenzymes. In contrast, genomes of diazotrophic, proteobacterial model endophytes such as *Azoarcus olearius* BH72^49^, *Klebsilla pneumoniae* 342^53^, or *Methylobacterium populi* BJ001^54^, harboured a much smaller gene density (2,3%, 2.1%, or 2.3%, with 11–23 predicted signal peptides, respectively). The rice *Verrucomicrobia* appear to participate also in several steps of nitrogen cycling (Supplementary Note 2, Supplementary Table S7).

Combining culture dependent (Supplementary Tables S6,S8) and –independent data (Supplementary Tables S6,S7), we evaluated further possible plant-host related characteristics of our strains. Two-component chemosensory systems including putative methyl-accepting proteins (MCP) and chemotaxis proteins (Che) were encoded in all four genomes. These proteins have an important role for root colonization by movement toward root exudate components^55^. An alternative bacterial movement on surfaces through twitching motility^56^, and also adherence to the plant host^57^, can be mediated by type IV pili. Several components for the pilus apparatus were encoded in all four genomes, particularly PilT which is responsible for retraction of the pilus in twitching motility and essential for endophytic rice root colonization by *Azoarcus olearius* BH72^58^. In addition, our isolates carried genes for agglutination proteins like hemagglutinin or putative adhesins. Also Type III secretion systems which commonly secrete effector proteins into host cells were traced in the genomes of the good colonizers EW11 and LW23 (Supplementary Note 2).

**Figure 4:**
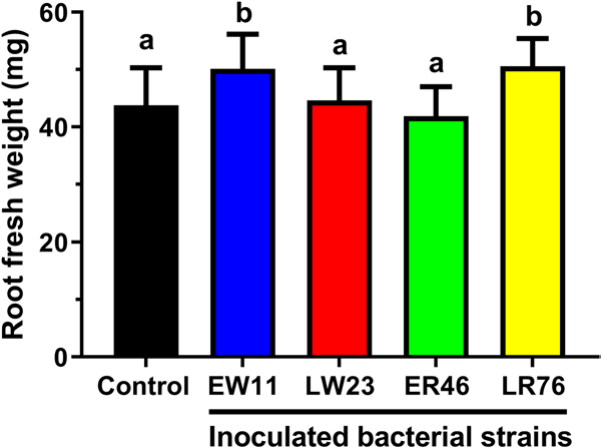
Root growth promotion by verrucomicrobial isolates. Sterile seedlings of *O. sativa* cv. Nipponbare were grown in hydroponic culture without (control) or with inoculation of single verrucomicrobial strains, and root fresh weight was evaluated 7 d post inoculation. Error bars above columns indicate standard deviations from three independent experiments (6–12 plants each). Columns headed by different letters are significantly different from each other at P < 0.05 according to analysis of variance (ANOVA) followed by uncorrected Fisher’s LSD.

Particularly significant is the detection of traits for plant growth promotion in our isolates (Supplementary Tables S6,S7). Bacterial synthesis of plant hormones such as the auxin indole-3-acetic acid (IAA) may increase root development. Hints for either weak or strong synthesis of indole-related compounds, such as IAA (indole acetic acid) and its precursors IAM (indole-acetamide) or IPA (indole-3-pyruvic acid)^59^, were detected for all strains in a colorimetric bioassay. Phosphate is often a limiting factor in soil^60^. Mineralization or solubilisation by bacteria can occur through the production and secretion of organic acids or phosphatases^61,62^. All strains showed activity of acid phosphatases for the solubilisation of organic phosphates^63^, and possessed also genes for citrate synthase; two strains were capable of solubilizing phosphate in a plate assay based on tricalcium phosphate (Supplementary Tables S6,S7), however this test is only indicative of phosphate solubilisation in soils and does not prove a direct contribution of phosphorous to the plant^64^. In order to obtain hints whether root-associated verrucomicrobia can indeed affect plant growth, short-term inoculation assays (7 d) were carried out for rice seedlings grown under gnotobiotic conditions. In comparison to the sterile, uninoculated control, an endophytic (ER46) and a surface strain (LR76) originating from *O. longistaminata* increased fresh weight of roots (Fig. 4), which was not significantly affected by strains LW23 or EW11 under these conditions.

## Concluding Remarks

There is a lack of fundamental knowledge on principles underlying assembly of a complex root microbial community and its interplay with hosts for plant fitness. Defined collections of plant microbes will help to address these questions in reconstitution experiments under sterile conditions, however current collections like for *Arabidopsis thaliana* that cover roughly 60% of the root community^24^ or other collections^25^, miss *Verrucomicrobia* entirely. Endophytes, that deeply ingress into the tissue and are probably most tightly associated with plants, have previously never been cultivated among *Verrucomicrobia*. As *Verrucomicrobia* were recently estimated to account for up to 25% of rice root endophytes^21^, they are likely to play an important but hidden role in the root interactome. Thus, our novel root-associated isolates provide a valuable new resource to disclose functions of these slow-growing, but often highly abundant bacteria in complex root microbiota.

**Figure 5:**
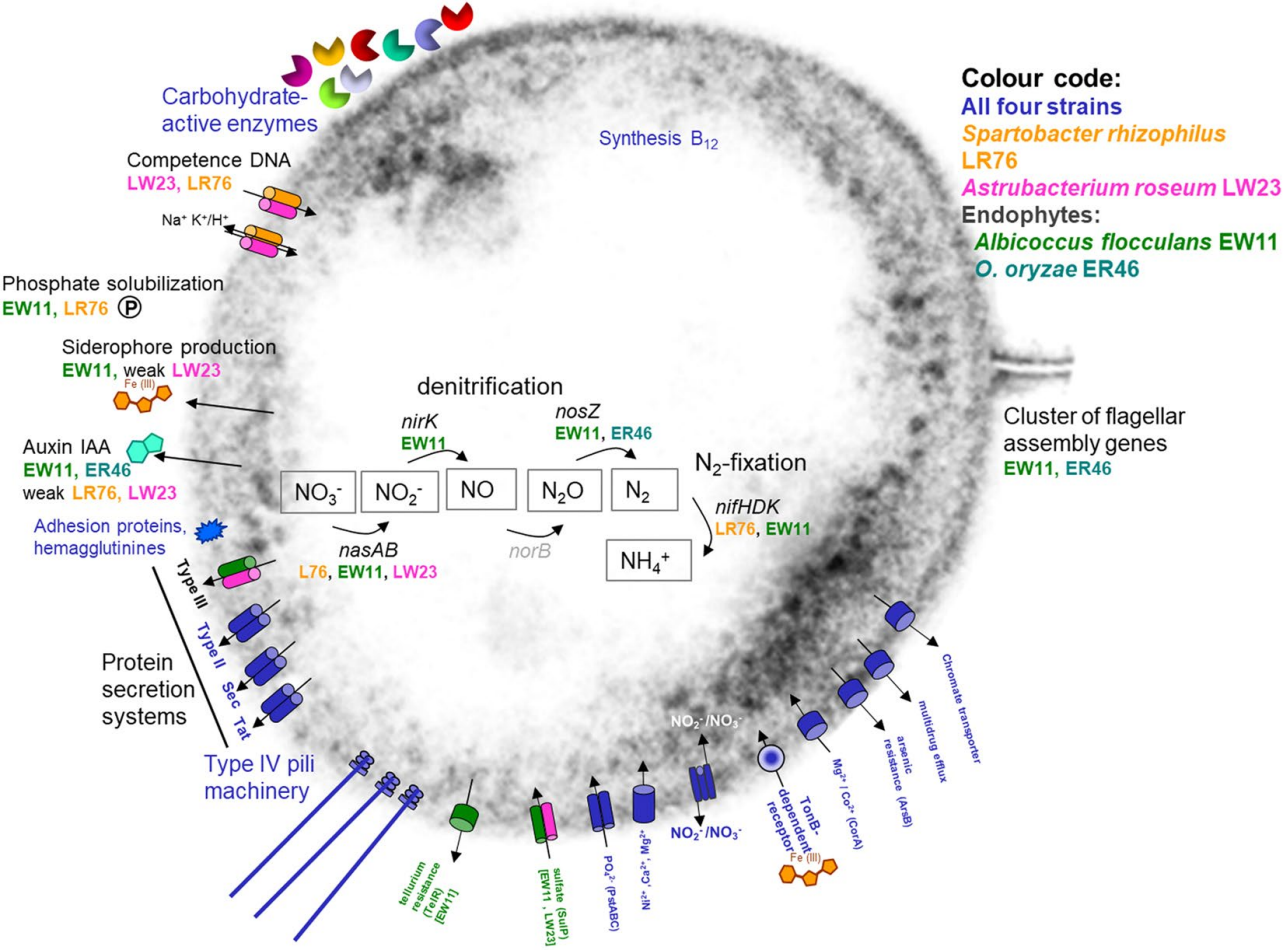
Overview of selected features of plant-colonizing *Verrucomicrobia* strains based on genomic data or experimental tests. Features common to all four isolates are shown in blue, colour codes for occurrence in *Spartobacter rhizophilus* LR76, *Astrumicrobium roseum* LW23, or endophytes *Albicoccus flocculans* EW11 or *Opitutus terrae* ER46 are given in the figure. In addition to carbohydrate metabolism, selected transporters and nitrogen cycling pathways, features likely related to plant-microbe interactions are shown.

Despite their phylogenetic divergence from commonly known root bacteria, *Verrucomicrobia* share a combination of characteristics typical for other beneficial root microbes that were previously unknown to *Verrucomicrobia*^15^ (Fig. 5). Comparative analysis of signature proteins did not indicate a recent gene transfer to our plant colonizers, consistent with an ancient origin of bacterial plant colonization. This enabled us to define deep-rooting core features across phyla for association with plants, and thus gives fundamental insights into functional adaptations to the specific underground niches provided by the earth’s most important primary producers, plants.

## Materials and Methods

### Bacterial isolation and cultivation conditions

Rice grains (*Oryza sativa ssp. japonica* cv. Nipponbare and *O. longistaminata*) were dehusked, surface sterilized, washed and germinated as described previously^65,66^. After germination, the plants were grown in moistened rice paddy soil obtained from Vercelli (Italy) in a phytotron (30 °C, 80% humidity, 14 h/10 h day/night cycle at 16 000 lx). The plants were irrigated with distilled water regularly. Root systems from 2 – 10 cm soil depth were taken from 8 week-old plants, washed with tap water, and shaken in wash buffer (SM-medium^46^) without carbon and nitrogen source) together with sterile glass beads (Ø 1 mm, Roth, Karlsruhe, Germany) in Erlenmeyer flasks on a reciprocal shaker^46^, to obtain the rhizoplane bacteria in the supernatant.To obtain putative endophytic bacteria, washed roots were surface-sterilized by shaking 2 min in 5% sodium hypochlorite solution and five washing steps with sterile water^67^. To check for surface contamination, the samples were dipped on R2A medium^68^ agar plates and incubated as described below, and no colonies were visible after incubation. Surface-sterilized roots were homogenized with sterile quartz sand in a sterile mortar. Serial dilutions were plated on R2A agar supplemented with vitamin solution^69^, and incubated at 28 °C.

### Amplification of 16S rDNA sequence and phylogenetic analysis

As template for the amplification of 16S rRNA gene (rDNA) sequences, 2 µl of bacterial cell lysate in a 50 µl reaction was used. To obtain the lysate, cells were incubated in 1 X Tris-EDTA buffer pH 8.0 containing 0.01% Tween20 [v/v] at 95 °C for 15 min. PCR amplification of almost complete 16S rDNA genes was performed using 2.5 U *Taq* Polymerase (Thermo Fisher Scientific, Waltham, Massachusetts, USA), 50 µM of each dNTP and the primer pair Bac8uf (5′-AGAGTTTGATNHTGGYTCAG-3′) and Univ1492r (5′-GGNTCCTTGTTACGACTT-3′)^70,71^ at 95 °C for 5 min, 40 cycles of 95 °C for 1 min, 52 °C for 30 sec, 72 °C for 2 min and a final elongation of 10 min at 72 °C in a Biometra thermocycler. Sequencing of PCR amplicons was carried out at the LGC Genomics Sequencing Service (Berlin, Germany). Quality of the sequences obtained was checked manually using BioEdit 7.2.5^72^. Sequences were aligned using a secondary- structure based Infernal aligner obtained through the Ribosomal Database project^73^. Alignments were reviewed in MEGA 7^74^ and if necessary manually refined. Phylogenetic trees were based on 1228 positions and were reconstructed by the maximum likelihood method implemented in IQ-TREE^75^ under the best-fit model (GTR + F + R6) according to the Bayesian information criterion (BIC) model finder.

Whole-genome-based phylogenetic comparisons were done with a composition vector approach using CVTree3^76,77^ with a K’mer size of 6 (CVTree3, http://cvtree.big.ac.cn/cvtree/cvtree/).

### Tests of growth characteristics

All tests of growth characteristics were carried out at 28 °C under aerobic conditions, except for temperature tolerance or anaerobic growth, respectively. Tolerance to extreme pH conditions was tested in R2A medium adjusted to pH 4 or 5 with homopiperazine-1,4-bis(2-ethanesulfonic acid) (Homo-Pipes) as buffer system. For alkaline conditions at pH 8.5 or 9.5, 3-(cyclohexylamino)-2-hydroxy-1-propanesulfonic acid (CAPSO) buffer was used. Cultures were incubated at 28 °C until growth for positive controls on neutral R2A agar became visible.

Anaerobic growth was tested on agar plates incubated for 14 days at 28 °C in an anaerobic reaction chamber (Merck Millipore, Darmstadt, Germany) supplemented with GasPak indicator stripes (Becton, Dickinson and Company, New Jersey, USA) and Oxoid AneroGen Sachets (Thermo Fisher Scientific, Waltham, Massachusetts, USA).

### Testing plant growth promotion characteristics and colonization competence

For the evaluation of inorganic phosphate solubilisation, Pikovskaya agar plates were used^78^. Bacteria were grown in liquid culture before transfer to the plates in droplets. Incubation was done at 28 °C for 7 days. The formation of clear halo around the bacterial droplets was evaluated, with *Bacillus subtilis* as positive control. Production of siderophores was tested according to^79^ on Chrome azurol S agar plates with modifications. Chrome azurol S agar plates without piperazine and deferrization were overlaid with a solid layer of R2A medium, before transferring droplets of bacterial culture. A formation of orange halos around the bacterial droplets was rated as positive for siderophore production, with *Azospirillum brasilense* Sp7 as positive control. The survey for indole-3-acetic acid (IAA) production and indole-related compounds was performed according to^80^ after growth in presence of _L_-tryptophan. Briefly, the cultures as well as the positive control (*A. brasilense* Sp7) were grown in liquid R2A Medium supplemented with 6 mM _L_-tryptophan. After centrifugation, the supernatant was incubated with salkowski reagent (0.01 M FeCl_3_ in 35% perchloric acid) in the dark at RT, and a colour change from yellow to red with an absorbance peak at 530 nm indicated a positive result.

For evaluation of colonization competence and plant growth promotion, *O. sativa* cv. Nipponbare was grown in a hydroponic test system. Dehusked rice grains were surface-sterilized, washed and germinated in magenta vessels (Sigma-Aldrich, Darmstadt, Germany) as described above. Instead of transfer to pots, the seedlings were grown in sterile glass tubes (100 ml) containing stainless-steel mesh as carrier, in 50 ml plant medium without nitrogen source (as described in^65^). Iron-citrate in the medium was replaced by iron-EDTA. Bacteria were grown in liquid R2A medium or R2A agar at 28 °C, washed with plant medium and inoculated to OD 0.067. After incubation in the phytotron for 7 days, roots were inspected for colonization, and plant medium was streaked on R2A agar to detect possible microbial contaminants. In three separate experiments on plant growth promotion, root fresh weight was determined after 7 days.

### Microscopy

For evaluation of root colonization, roots were stained at room temperature with SYBR Green I nucleic acid stain (Sigma-Aldrich, Darmstadt, Germany) in a 1000x working solution. For detection of cellular compartments, 500x working solution was applied. Fluorescence of the root material was evaluated using an epifluorescence microscope (Zeiss Axioplan 2, Carl Zeiss AG, Oberkochen, Germany) with a Zeiss Axiocam 503 colour camera. For detection of green fluorescence at 522 nm, the Zeiss filter set 09 was used. To visualize the plant auto-fluorescence, the Zeiss filter set 43 He was used.

For imaging by confocal laser scanning microscopy (CLSM), a Zeiss 880 Microscope (Carl Zeiss, Jena, Germany) with the high resolution Airyscan detector was used, equipped with an argon/neon-laser with a wavelength λ = 488, 543 nm. A 20× dry objective lens (Zeiss NEONFLUAR 20×, 0.5 HD) and 40x oil objective lens (Zeiss NEONFLUAR 40×, 0.5 HD) were used. For the excitation and emission the following settings were used: 488 nm laser (100 mW) and beam splitters BP495-575 for the green dye; 543 nm laser (100 mW) and beam splitters BP570-650 for auto-fluorescence. The images were reconstructed using ZEN software (Black Edition, 2011, Carl Zeiss, Germany) based on the structured illumination algorithm. Further analysis was performed on reconstructed super-resolution images in ZEN software blue edition (2012, Carl Zeiss, Jena, Germany). 3D images of the infected roots were constructed from the Z-stack images. The z-stack ZEN file was first exported as multi TIFF files, and then the multiple TIFF files were converted to a single 3D image by ImageJ (ImageJ bundled with 64-bit Java 1.8.0_112).

Before transmission electron microscopy cells were grown under micro-aerobic conditions (for strains LR76, EW11 and ER46) or under aerobic conditions (strain LW23) in liquid R2A medium (supplemented with vitamins, and fructose and maltose at 0.5 g/l). Cells were fixed in 2% glutaraldehyde in cacodylate buffer (50 mM, pH 7.2) for 45 minutes on ice followed by washing in cacodylate buffer and HEPES buffer (50 mM, pH 7.2). The cells were then embedded in agarose blocks and dehydrated in increasing concentrations of ethanol at −20 °C before stepwise infiltrations in LR White resin (Sigma Aldrich, Missouri, USA)^65^. Agar blocks were transferred to gelatine-capsules before polymerization at 65 °C for 48 hours. Ultra-thin sections were prepared with an Ultramicrotome Ultracut E (Reichert-Jung, Leica, Wetzlar, Germany) and stained on grids with filtered uranyl acetate in distilled water (3%) followed by lead citrate in CO_2_-free distilled water (2.5%) for 1–2 minutes, respectively. Sections were viewed in a Zeiss 900 A Transmission Electron Microscope (Carl Zeiss AG, Oberkochen, Germany) operated at 80 kV.

### Draft Genome sequencing and bioinformatic analyses

For genomic DNA extraction bacterial cell pellets were washed in Tris-EDTA Saline (TES, pH 8) buffer and DNA was isolated by using a phenol/chloroform extraction protocol^81^. DNA was dissolved in TE buffer and stored at −80 °C. DNA concentration was determined with a NanoDrop 2000 spectrophotometer. Library preparation was performed as described by the manufacturer using the TruSeq PCR-Free Library Preparation Kit (Illumina, San Diego, California, USA) after mechanical fragmentation of the genomic DNA into 550 bp fragments by application of the Covaris M220 focused-ultrasonicator (ThermoFisher Scientific, Waltham, Massachusetts, USA). The bacterial genomes were sequenced (2 × 300 bp) using an Illumina MiSeq platform (Illumina, San Diego, California, USA). We assembled the sequencing reads for each of the four genomes using the CLC Genomics Workbench 9.0 *De Novo* Assembly 1.3 with default settings (Qiagen, Venlo, Netherlands). After assembly, contig sequences with a minimum size of 1 kb were included for further analyses.

Draft genomes of the described strains were annotated by the Rapid Annotation tool using the Subsystems Approach (RAST) Server^82,83^ and compared to results of the annotation derived by the NCBI prokaryotic genome annotation pipeline (PGAP)^84^. Functional annotation and automatic pathway reconstruction were generated by the Kyoto Encyclopaedia of Genes and Genomes (KEGG) Automatic Annotation Server (KAAS) by GHOSTX comparisons^85,86^. PFAM domain searches were carried out with CLC Genomics Workbench version 11.0 using the Pfam-A family database (version 31.0) and profile’s gathering cutoff. The abundance of Pfam domains in bacteria from the three compartments was compared by multiple T-tests not assuming a consistent standard deviation using GraphPad Prism v7.05 (Graphpad software). This software was also used for one-way ANOVA (analysis of variance) followed by Holm-Sidak’s multiple comparison test. P > 0.05 was considered not significant. Venn diagrams were drawn with unique Pfam domain accessions using a Venn diagram drawing tool (http://bioinformatics.psb.ugent.be/webtools/Venn/). To visualize the relationship between protein domain group means of the three compartments, a between-group principal component analysis (bgPCA) was carried out. Protein domain differences between compartments was assessed by non-parametric multivariate analysis of variance (PERMANOVA) based on 9999 permutations using a Bray-Curtis similarity index. Multivariate analysis was performed with Past 3^87^.

Carbohydate-active enzymes were identified in the dbCAN2 annotation tool^52^ using HMMER search at E-Values < 1e-15, coverage >0.35.

The annotated complete genome sequences were deposited in GenBank: strain LW23 (Accession No. QAZA00000000), strain LR76 (Accession No. QAYZ00000000), strain EW11 (Accession No. QAYY00000000) and strain ER46 (Accession No. QAYX00000000).

## Supplementary Information

Supplementary Information.

Supplementary Video 1.

Supplementary Video 2.

Supplementary Video 3.

Supplementary Video 4.

Supplementary Table S4.

Supplementary Table S5.
