# Supplementary material for: Novel cultivated endophytic Verrucomicrobia reveal deep-rooting traits of bacteria to associate with plants
Source: Sci Rep. 2020 May 26;10:8692. doi: 10.1038/s41598-020-65277-6 (PMC7251102; doi:10.1038/s41598-020-65277-6)
Supplement: Supplementary file 1 — Supplementary Information. [file 41598_2020_65277_MOESM1_ESM.pdf]

Supplementary information

Bünger et al.

Novel cultivated endophytic Verrucomicrobia reveal deep-rooting traits of bacteria to associate with plants

Wiebke Bünger[1], Jiang Xun[1], Jana Müller [1,2], Thomas Hurek[1] and Barbara Reinhold-Hurek[1*]

[1] *Department of Microbe-Plant Interactions, University of Bremen, Bremen, Germany*

[2] *Current address: Department of Botany, University of Bremen, Bremen, Germany*

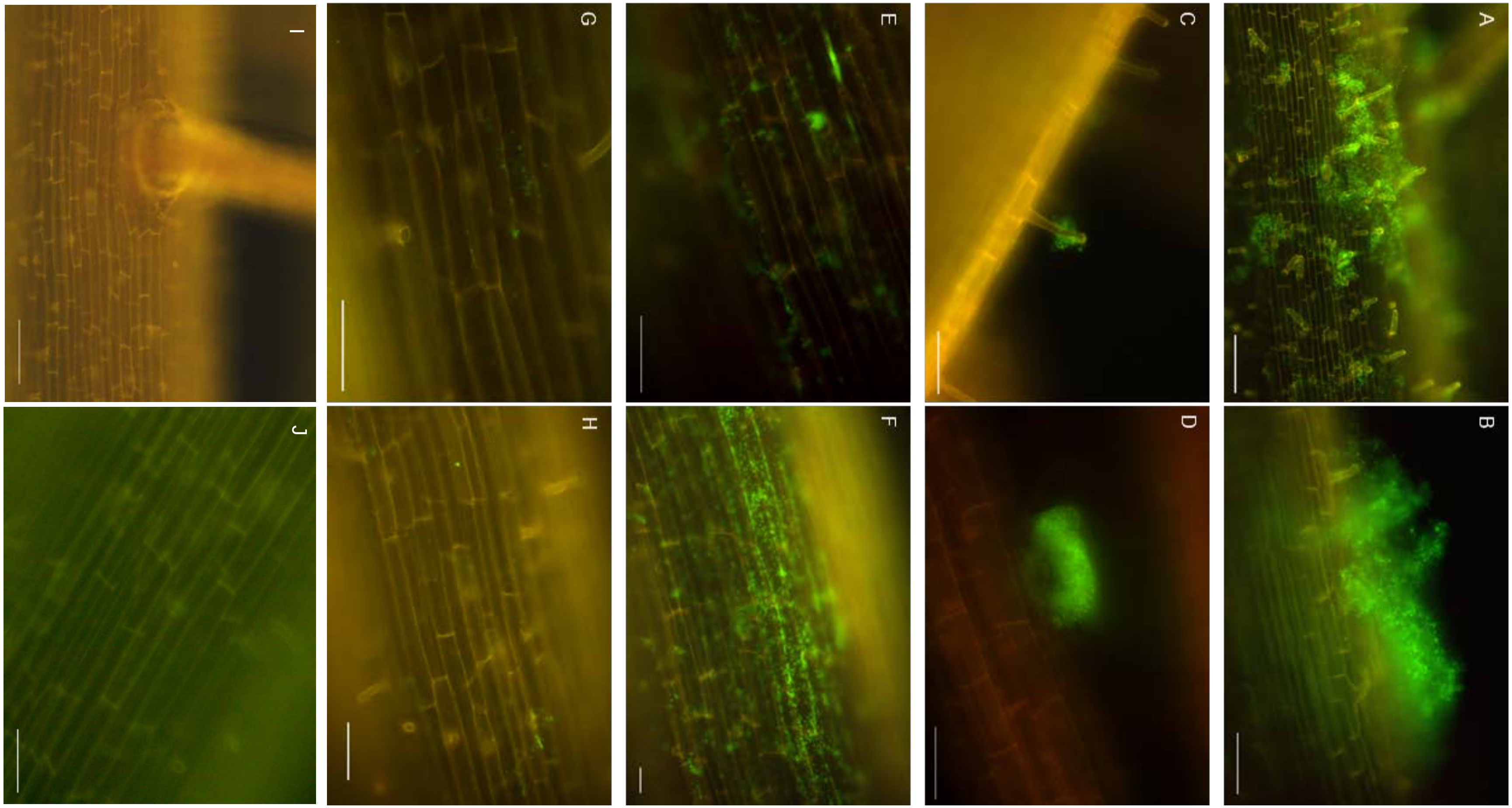

Not inoculated

Opitutus oryzae strain ER46

Albicoccus flocculans strain EW11

Astrumicrobium roseum strain LW23

Spartobacter rhizophilus strain LR76

Supplementary Figure 1 Fluorescence microscopy of *O. sativa* cv. Nipponbare roots 8 dpi after inoculation in hydroponic gnotobiotic culture. Roots were incubated with SYBR® green to visualize bacteria. **A, B,** Colonization of strain LR76; **C, D,** colonization of strain LW23; **E, F,** colonization of strain EW11; **G, H,** colonization of strain ER46; **I, J,** uninoculated control, not contaminated by microbes. Bars indicate 100µm (A, I), 50 µm (B,C,D,E,G,H, J) and 20 µm (F).

Supplementary Note 1 – Placement of strain LW23 in novel subdivision 8

The following data suggest that strain LW23 belongs to a novel subdivision 8: (i) Methanotrophic strains (*Methylacidimicrobium tartaophylax*, *M. cyclophantes* and *M. fagopyrum*) from volcanic soil [1] in subdivision 6 were only distantly related to it according to phylogenetic reconstruction of almost complete 16S rDNA sequences (85-86 % sequence identity, supported by high bootstrap values) (Fig. 1A). (ii) Genome sizes and G+C contents in subdivision 6 strains (*Methylacidiphilum infernorum* V4 and *M. fumariolicum* strain SolV) were much lower than for strain LW23 (Supplementary Table S1. The genome sizes of the methanotrophic strains (*Methylacidiphilum infernorum* V4 and *M. fumariolicum* strain SolV) belonging to subdivision 6 neighbouring to strain LW23, were with a total length of 2.3 and 2.4 Mb at the lower range of this phylum, suggesting limited potential for metabolic and life style diversity. Also the G+C contents of both genomes were low (40.9 % and 45.5 %) [2,3] compared to strain LW23. Although *Verrucomicrobium* sp. strain GAS474 has a higher G+C content of 65.8% than methylotrophs and no methane monooxygenase gene, the genome size is also small (3,7 Mb) [4]. (iii) Physiological characteristics of the methanotrophic strains with low pH (1-3.5) and high temperature optima [1] did not correlate with growth characteristics of strain LW23 (Supplementary Table S2). (iv) Metagenomes of verrucomicrobia identified in a brackish microbiome (*Verrucomicrobia* BACL9 MAG) were also not related to LW23, because they clustered as a sister lineage of subdivision 6 [5] or were proposed as a novel subdivision according to concatenated household protein sequences [6]. They cluster with environmental sequences according to 16S rRNA gene phylogeny as second sister lineage of methylotrophs (Fig. 1A). (v) To reveal phylogenetic relationships with an independent whole-genome-based method, CVTree [7] was applied that uses peptide patterns, with K-values of 5–6 being most suitable for prokaryotes [7]. Verrucomicrobial subdivisions were well-separated from each other (Fig. 1B), and members of both lineages of methylotrophs were placed in the same subdivision 6. In contrast, isolate LW23 was only distantly related to them but clustered with *Verrucomicrobiaceae* bacterium GAS474 placed with methylotrophs in 16S rRNA analysis. This isolate from forest soil had been proposed as a novel branch [4], but shared the small genome size with methylotrophs despite a relatively high G+C content (Supplementary Table S1). (vi) Genomes of both isolates did not harbour typical genes of methanotrophs encoding AmoA. Therefore, we propose description of a novel subdivision 8 that includes a large number of environmental bacteria, strain LW23 and probably strain GAS474.

The following data suggest that *Astrumicrobium roseum* strain LW23 belongs to a novel subdivision 8: (i) Methanotrophic strains (*Methylacidimicrobium tartaophylax*, *M. cyclophantes* and *M. fagopyrum*) from volcanic soil [1] in subdivision 6 were only distantly related according to phylogenetic reconstruction of almost complete 16S rDNA sequences (85-86 % sequence identity, supported by high bootstrap values) (Fig. 1A). (ii) Genome sizes and G+C contents in subdivision 6 strains (*Methylacidiphilum infernorum* V4 and *M. fumariolicum* strain SolV) were much lower (Supplementary Table 1). (iii) Physiological characteristics of the methanotrophic strains with low pH (1-3.5) and high temperature optima [1] did not correlate with growth characteristics of strain LW23 (Supplementary Table 2). (iv) Metagenomes of verrucomicrobia identified in a brackish microbiome (Verrucomicrobia BACL9 MAG) clustered as a sister lineage of subdivision 6 [5] or were proposed as a novel subdivision according to concatenated household protein sequences [6]. They cluster with environmental sequences according to 16S rRNA gene phylogeny as second sister lineage of methylotrophs (Fig. 1A). (v) To reveal phylogenetic relationships with an independent whole-genome-based method, CVTree [7] was applied that uses peptide patterns, with K-values of 5–6 being most suitable for prokaryotes [7]. Verrucomicrobial subdivisions were well-separated from each other (Figure 1B), and members of both lineages of methylotrophs were placed in the same subdivision 6. In contrast, isolate *Astrumicrobium roseum* LW23 was only distantly related to them but clustered with *Verrucomicrobiaceae* bacterium GAS474 placed with methylotrophs in 16S rRNA analysis. This isolate from forest soil had been proposed as a novel branch [4], but shared the small genome size with methylotrophs despite a relatively high G+C content (Supplementary Tables S1, S3). (vi) Genomes of both isolates did not harbour typical genes of methanotrophs encoding AmoA. Therefore, we propose description of a novel subdivision 8 that includes a large number of environmental bacteria, strain LW23 and probably strain GAS474.

Supplementary Note 2 – Some physiological and plant-microbe-interaction related traits of verrucomicrobial isolates

Nitrogen cycling

Microbial nitrogen cycling is instrumental for fertile soils and plant growth, and the new *Verrucomicrobia* may participate in several steps. The capability for nitrogen fixation was detected in only few cultivated members of the *Verrucomicrobia*, although their nitrogenase (*nifH*) genes are common in many habitats [8-10]. Only strains EW11 and LR76 were found to be equipped with the *nifHDK* cluster essential for nitrogen fixation, but did not possess the alternative *anfHDK* cluster. Most or all strains harboured genes for nitrate/nitrite transport and assimilation, and parts of denitrification pathway (Supplementary Table S7). Strains LW23 and ER46 possessed valine and leucine arylamidase (Supplementary Table S8), probably involved in the release of amino acids from soil organic matter.

Protein secretion systems

Bacterial protein secretion systems are involved in transport of enzymes into the environment or for the interaction with other prokaryotic or eukaryotic cells. Like in many other sequenced *Verrucomicrobia*, evidence for components of the Twin-arginine translocation (Tat) and Type II secretion pathway, the (Sec-SRP pathway) was found in all four genomes (Supplementary Table S7). In addition, two strains LW23 and EW11 harboured genes for the Type III secretion system, unusual for most other sequenced *Verrucomicrobia* (Supplementary Table S7). As it commonly secretes effector proteins into host cells, it is likely linked to the plant-associated lifestyle.

References:

1. van Teeseling, M. C. F. *et al.* Expanding the Verrucomicrobial Methanotrophic World: Description of Three Novel Species of *Methylacidimicrobium* gen. nov. *Appl. Environ. Microbiol.* **80**, 6782-6791 (2014).
2. Hou, S. B. *et al.* Complete genome sequence of the extremely acidophilic methanotroph isolate V4, *Methylacidiphilum infernorum*, a representative of the bacterial phylum *Verrucomicrobia*. *Biol Direct* **3** (2008).
3. Khadem, A. F. *et al.* Draft Genome Sequence of the Volcano-Inhabiting Thermoacidophilic Methanotroph *Methylacidiphilum fumariolicum Strain SolV*. *J. Bacteriol.* **194**, 3729-3730 (2012).
4. Pold, G. *et al.* Genome sequence of *Verrucomicrobium* sp. strain GAS474, a novel bacterium isolated from soil. *Genome Announc.* **6** (2018).
5. He, S. *et al.* Ecophysiology of freshwater Verrucomicrobia inferred from metagenome-assembled genomes. *mSphere* **2** (2017).
6. Hugerth, L. W. *et al.* Metagenome-assembled genomes uncover a global brackish microbiome. *Genome Biol* **16**, 279 (2015).
7. Zuo, G. H. & Hao, B. L. CVTree3 Web Server for whole-genome-based and alignment-free prokaryotic phylogeny and taxonomy. *Genom. Proteom. Bioinf.* **13**, 321-331 (2015).
8. Penton, C. R. *et al.* *NifH*-harboring bacterial community composition across an Alaskan permafrost thaw gradient. *Front Microbiol* **7**, 1894 (2016).
9. Kox, M. A. R. *et al.* Effects of nitrogen fertilization on diazotrophic activity of microorganisms associated with *Sphagnum magellanicum*. *Plant Soil* **406**, 83-100 (2016).
10. Wang, J., Bao, J. T., Li, X. R. & Liu, Y. B. Molecular ecology of *nifH* genes and transcripts along a chronosequence in revegetated areas of the tengger desert. *Microb. Ecol.* **71**, 150-163 (2016).

Supplementary Tables Bünger et al.

Supplementary Table S1. General features of draft genomes[a] of *Verrucomicrobia* isolates according to the results obtained from the PGAP.

Feature	Strain and subdivision			
	Strain LR76	Strain LW23	Strain EW11	strain ER46
	Subd. 2	Subd. 8	Subd. 4	Subd. 4
% mol GC	60.7	61.7	63.6	67.1
Size (Mb)	4.8	6.6	6.4	5.7
Contigs (>1 Kb)	16	56	45	25
Coding genes	4170	4879	5002	4256
tRNAs	49	56	46	45
Accession Number	QAYZ00000000	QAZA00000000	QAYY00000000	QAYX00000000

[a] After assembly, the draft genomes were annotated by RAST [1-3], and results were compared with data derived by the NCBI prokaryotic genome annotation pipeline (PGAP) [4]. Metabolic pathways were revised by the KEGG Automatic Annotation Server (KAAS (Moriya *et al.*, 2007)) and metabolic pathway mapper. It was found that some annotations were unique to the RAST assignments and not identified by the PGAP. Generally, small differences in annotation results obtained from different pipelines are common [5].

1. Aziz, R. K. *et al.* The RAST server: Rapid annotations using subsystems technology. *BMC Genomics* **9**, 1-15 (2008).
2. Overbeek, R. *et al.* The SEED and the Rapid Annotation of microbial genomes using Subsystems Technology (RAST). *Nucleic Acids Res.* **42**, D206-D214 (2014).
3. Brettin, T. *et al.* RASTtk: A modular and extensible implementation of the RAST algorithm for building custom annotation pipelines and annotating batches of genomes. *Sci. Rep.* **5** (2015).
4. Tatusova, T. *et al.* NCBI prokaryotic genome annotation pipeline. *Nucleic Acids Res.* **44**, 6614-6624 (2016).
5. Kisand, V. & Lettieri, T. Genome sequencing of bacteria: sequencing, de novo assembly and rapid analysis using open source tools. *BMC Genomics* **14**, 211 (2013).

Supplementary Table S2. Morphological and physiological characteristics of *Verrucomicrobia* isolates

Characteristic	*Strain*			
	Strain LW23	Strain LR76	Strain EW11	Strain ER46
Isolation source	Rhizoplane *Oryza longistaminata*	Rhizome surface *O. longistaminata*	Endosphere Root *O. sativa*	Endosphere Rhizome *O. longistaminata*
Cell size	3-4 µm long, 1 µm in diameter	2-3 µm long, 0.7 µm in diameter	2-3 µm	1.5-2 µm
Cell shape	rod	rod	cocci	cocci
Colony color	pink	opaque yellow	pale yellow	white
Motility	-[a]	-	+	+
Growth temperature [°C][b]	28	28	28	28
Optimum range	20-37	28-37	28-40	28-37
pH for growth	7.0	7.0	7.0	7.0
Optimum range	5.0-8.5	6.5-8.0	6.0-8.5	6.0-8.5

[a] Abbreviations: + positive, - negative
[b] All strains grown aerobically

Supplementary Table S3. ANI values between draft genome sequences of strains LW23, LR76, EW11 and ER46 and respective reference strains calculated by JSpecies[1] as well as and 16S rRNA gene identities to most closely related isolates

Reference strain	Isolate			
	Strain LR76	Strain LW23	Strain EW11	Strain ER46
	ANI (%) or 16S rRNA gene identity (%)			
**ANI value**				
Terrimicrobium sacchariphilum NM-5	86.59 [a]			
(Subdivision 2)	89.00 [b*]			
Opitutus terrae PB90-1			70.08 [a]	72.01 [a]
(Subdivision 4)			83.24 [b*]	83.53 [b*]
Methylacidiphilum infernorum V4		63.21 [a*]		
(Subdivision 6)		87.15 [b*]		
Verrucomicrobiaceae bacterium GAS474		68.07 [a*]		
(Probably Subdivision 8)		82.53 [b*]		
Chthoniobacter flavus Ellin 428			65.66 [a*]	65.89 [a*]
(Subdivision 2)			82.27 [b*]	82.33 [b*]
**16S rRNA gene identity** [c]				
Terrimicrobium sacchariphilum NM133878	99			
Verrucomicrobia sp. *TSB47*			95	
Opitutus sp. *VeSm13*				99
No close relative		-		

[a] ANIb

[b] ANIm

[*] Suspicious alignment reported by Jspecies

[c] For strain LR76, *Terrimicrobium sacchariphilum* was the closest cultured relative at a 16S rRNA gene sequence identity of 99 %. The DNA G+C content for *T. sacchariphilum* is 46.3 mol %, in contrast to 60.7 mol % for isolate L76 (Supplementary Table 1). Within species, differences in G+C content vary only between 1- 5 % [1,2]. Additionally, an ANI value of less than 90% gave strong support for at least a distinct novel species, or rather a new genus based on the large genomic and physiological differences. For strain EW11, the closest cultured relative was *Verrucomicrobia* sp. TSB47 (16S rRNA gene sequence identity 95 %). Since the 16S rRNA gene sequence identity between the two strains was below 98.7-99 %, a threshold value for 16S rDNA gene sequence identity for species differentiation, strain EW11 represents at least a new species within subdivision 4 with a new genus name (as values < 94.5 % suggest distinct genera,

no named cultivated relative). Preliminary novel names: **Strain EW11**: *Albicoccus flocculans* gen. nov. (Al.bi.coc'cus. L. adj. albus, white; N.L. masc. n. coccus, from Gr. masc. n. kokkos, a berry, coccus; N.L. masc. n. *Albicoccus*, white coccus); sp. nov. (floc'cu.lans. N.L. masc. Part. Adj. flocculans, flocculating). **Strain LR76**: *Spartobacter rhizophilus* gen. nov. (Spar.to.bac'ter. related to *Spartobacteria*, N.L. masc. n. *bacter*, rod); sp. nov. (rhi.zo.phi'lus. Gr. n. rhiza root; Gr. adj. philos loving; N.L. masc. adj. *rhizophilus* root-loving). **Strain LW23**: *Astrumicrobium roseum* gen. nov. (As.tru.mi.cro'bi.um. L. neut. n. astrum, star; N.L. neut. n. microbium, a microbe; N.L. neut. n. *Astrumicrobium*, star-like microbe referring to star-like aggregates); sp. nov. ro'se.um. L. adj. roseus, rose-coloured, rosy). **Strain ER46**: *Opitutus oryzae* sp. nov. (o.ry'zae. L. fem. gen. n. *oryzae* of rice, pertaining to the habitat from which the strain was isolated).

1. Meier-Kolthoff, J. P., Klenk, H. P. & Göker, M. Taxonomic use of DNA G plus C content and DNA-DNA hybridization in the genomic age. *Int. J. Syst. Evol. Microbiol.* **64**, 352-356 (2014).
2. Rosselló-Mora, R. & Amann, R. The species concept for prokaryotes. *FEMS Microbiol. Rev.* **25**, 39-67 (2001).

Supplementary Table S4,

Supplementary Table S5

Provided as Exel-files

Supplementary Table S6. Plant growth promoting traits tested by laboratory bioassays compared to genomic data.

Characteristic	*Strains*			
	strain LW23	strain LR76	strain EW11	strain ER46
Phosphate solubilisation[b]				
Bioassay	-[a]	weak	+	-
pstA/B/C	+	+	+	+
IAA production				
bioassay	weak	weak	+	+
ipdC	-	-	-	-
Siderophore production/ reception				
bioassay	weak	-	+	-
fiu	+	-	+	-
Nitrogen fixation				
nifH	-	+	+	-

[a] +, indicates positive result for bioassay/presence of genes; -, indicates negative result.

[b] *Phosphate solubilisation:* All strains were tested positive for the activity of acid phosphatases in the APIzym assay, indicating a possible capability for the solubilisation of organic phosphates [1]. Also the production of organic acids, e.g. citric acid can be efficient in solubilizing mineral phosphates. All strains possessed genes encoding a citrate synthase (see also Supplementary Table S7). Genes encoding phosphate transporters (*pstABC*) were present in all draft genomes. The plate assay evaluating the capability to solubilize phosphate [2], only showed a positive result for strain EW11 and a weak result for strain LR76. However, also the other strains might be able to solubilize phosphate in their natural environment since phosphate solubilisation is highly complex and dependent on different factors like the nutritional and growth condition of the culture [3].

Auxin production: Among the plant-growth promoting traits, synthesis of plant hormones such as indole-3-acetic acid (IAA) as a predominant auxin can lead e.g. to increased root development. Several bacterial pathways for IAA biosynthesis are described so far. One important precursor for the synthesis is L-tryptophan, which is present in root exudates [4,5]. A colorimetric assay based on the reaction of iron(III)- chloride and perchloric acid in presence of

IAA formed from the added precursor L-tryptophan [6], showed different results for the tested isolates. Weak or strong reactions were detected for strain LR76 or strains EW11 and ER46, respectively. However, genes for the classical tryptophan-dependent IAA synthesis (*ipdC/ppdC* coding for phenylpyruvate decarboxylase and 3-pyruvate decarboxylase) were not found in either of the draft genomes.

Iron uptake: An universal chrome azurol S plate assay [7], which indicates the production of siderophores by a colour change, showed a positive or a weak reaction for strains EW11 and LW23, respectively. Genomic analyses indicated the presence of genes for TonB-dependent receptor proteins for all strains, see also Supplementary Table S7. The specific gene for a TonB-dependent siderophore receptor (*fiu*) was only found in strains LW23 and EW11

1. Walpola, B. C. & Yoon, M. H. Phosphate solubilizing bacteria: Assessment of their effect on growth promotion and phosphorous uptake of mung bean (*Vigna radiata* [L.] R. Wilczek). *Chil. J. Agr. Res.* **73**, 275-281 (2013).
2. Grönemeyer, J. L., Burbano, C. S., Hurek, T. & Reinhold-Hurek, B. Isolation and characterization of root-associated bacteria from agricultural crops in the Kavango region of Namibia. *Plant Soil* **356**, 67-82 (2012).
3. Reyes, I., Bernier, L., Simard, R. R. & Antoun, H. Effect of nitrogen source on the solubilization of different inorganic phosphates by an isolate of *Penicillium rugulosum* and two UV-induced mutants. *FEMS Microbiol Ecol* **28**, 281-290 (1999).
4. Spaepen, S., Vanderleyden, J. & Remans, R. Indole-3-acetic acid in microbial and microorganism-plant signaling. *FEMS Microbiol. Rev.* **31**, 425-448 (2007).
5. Teale, W. D., Paponov, I. A. & Palme, K. Auxin in action: Signalling, transport and the control of plant growth and development. *Nat. Rev. Mol. Cell. Bio.* **7**, 847-859 (2006).
6. Gordon, S. A. & Weber, R. P. Colorimetric estimation of indolacetic acid. *Plant Physiol.* **26**, 192-195 (1951).
7. Schwyn, B. & Neilands, J. B. Universal chemical assay for the detection and determination of siderophores. *Anal. Biochem.* **160**, 47-56 (1987).

Supplementary Table S7. Genes of *Verrucomicrobia* isolates potentially involved in plant-associated lifestyle.

	gene name	function of gene product	strain LW23	strain LR76	strain EW11	strain ER46
			locus tag (PGAP) or gene number (*RAST)			
**Phosphate solubilisation/ transport**						
	pstA	Phosphate transport system permease protein PstA	DB346_09115, DB346_23260, DB346_22255	DB345_01080	DB347_11985	DB354_00775
	pstB	Phosphate transport ATP-binding protein PstB	DB346_22260, DB346_09120	DB345_01075	DB347_11980	* 954
	pstC	Phosphate transport system permease protein PstC	DB346_09110, DB346_23255	DB345_01085	DB347_11990, DB347_13960	DB354_00770
		putative citrate synthase	DB346_14880	DB345_06810	DB347_05950	DB354_14025
**Iron transport/ siderophore recognition**						
	fiu	TonB-dependent siderophore receptor	* 1430	-	* 4576	-
	feoA	ferrous iron transport protein A	-	-	DB347_17325	DB354_04165
	feoB	ferrous iron transport protein B	-	DB345_14845	DB347_17330	DB354_04170
		iron permease	-	DB345_12970	-	-
		iron ABC transporter permease	DB346_08885	* 3637	-	-
	piuC	Iron-uptake factor PiuC	* 1431	-	-	-
	piuB	uncharacterized iron-regulated membrane protein; Iron-uptake factor PiuB	* 1432	-	* 4399	* 2650

	fhuB	Iron(3+)-hydroxamate import system permease protein FhuB	-	-	-	-
	fhuC	Iron(3+)-hydroxamate import ATP-binding protein FhuC	-	-	-	-
	fhuD	Iron(3+)-hydroxamate-binding protein FhuD, putative ferrichrome-iron TonB-dependent receptor	-	-	-	-
		putative TonB dependent receptor	DB346_01905, DB346_01800	* 42	DB347_01935, DB347_02030, DB347_00870, DB347_00100, DB347_23830, DB347_22840, DB347_19620, DB347_22840	DB354_16905, DB354_17490, DB354_19585
**Nitrogen cycle**						
	nasA	Nitrate reductase	* 4721	DB345_13250	DB347_11165, DB347_23785	-
	nirA	Ferredoxin-nitrite reductase	DB346_14580, DB346_16110	DB345_13260, DB345_10670	DB347_23800	DB354_14280
	nirB/D	Nitrite reductase (NADH)	* 3182	-	-	-
	nrfA/H	Nitrite reductase (cytochrome c-552)	-	-	DB347_22955, DB347_22960	DB354_02175, DB354_09800
	nirK	Copper-containing nitrite reductase	-	-	DB347_15580	-
	nosZ	Nitrous-oxide reductase	-	-	DB347_00340, DB347_00915	DB354_08355
	nifA	Nitrogenase (molybdenum-iron)-specific transcriptional regulator	-	* 3391	* 3334	-

nifN	Nitrogenase FeMo-cofactor scaffold and assembly protein	-	* 3383	* 3345	-
nifE	Nitrogenase iron-molybdenum cofactor biosynthesis protein NifE	-	DB345_11705	DB347_21930	-
nifB	Nitrogenase FeMo-cofactor synthesis FeS core scaffold and assembly protein	-	DB345_11695	DB347_21940	-
nifH	Nitrogenase (molybdenum-iron) reductase and maturation protein	-	DB345_11720	DB347_21910	-
nifD	Nitrogenase (molybdenum-iron) alpha chain	-	DB345_11715	DB347_21915	-
nifK	Nitrogenase (molybdenum-iron) beta chain	-	DB345_11710	DB347_21920	-
	putative nitrite/nitrate transporter	DB346_14560, DB346_14555, DB346_14565	DB345_15595, DB345_07080	DB347_10305, DB347_10855	DB354_14290
**Protein Transport**					
**Type II secretion system**					
gspF	general secretion pathway protein F	* 1894	* 37	DB347_13495, DB347_12230	DB354_17015
gspE	general secretion pathway protein E	* 663, 1893	DB345_16870, DB345_08675	DB347_14205, DB347_12225	DB354_19010, DB354_17415, DB354_21605
gspD	general secretion pathway protein D	* 2286	-	* 1335	* 2000
gspG	general secretion pathway protein G	DB346_09205	DB345_08630	DB347_05230	DB354_17365
**Type III secretion system**					

	yscC	Type III secretion outermembrane pore forming protein	DB346_19085	-	DB347_06325	-
	yscJ/ sctJ/ hrcJ/	Type III secretion protein J	DB346_15120	-	DB347_06295	-
	yscD	Type III secretion system inner membrane ring protein	DB346_23500			
	yscL/sctL	Type III secretion protein L	DB346_15130	-	DB347_06285	-
	yscN/ sctN/ hrcN	ATP synthase in type III secretion protein N	DB346_18795	-	DB347_06280, DB347_04295	DB354_19805
	yscQ/ sctQ/ hrcQ	Type III secretion protein Q	DB346_06190	-	DB347_06265	-
	yscR/ sctR/ hrcR	Type III secretion protein R	DB346_06185	-	DB347_06260	-
	yscS/ sctS/ hrcS	Type III secretion protein S	DB346_06180	-	* 3808	-
	yscT/ sctT/ hrcT	Type III secretion protein T	DB346_06175	-	DB347_06250	-
	yscU/ sctU/ hrcU	Type III secretion protein U	* 1271	-	DB347_06245	-
	yscV/ sctV/ hrcV	Type III secretion protein V	DB346_14435	-	DB347_06335	-
**Twin- arginine translocation (Tat) System**						
	tatA	sec-independent protein translocase protein TatA	DB346_20420	DB345_17585	DB347_22305, DB347_01085	DB354_05715
	tatC	sec-independent protein translocase protein TatC	DB346_19490	DB345_18035	DB347_13655, DB347_08050	DB354_05305
**Sec-SRP dependent**						

	secD/F	protein-export membrane protein SecD/F	DB346_19105	DB345_14240	* 4188	DB354_20180
	secE	preprotein translocase subunit SecE	DB346_08745	DB345_13310	DB347_05715	DB354_19010, DB354_13780
	secG	preprotein translocase subunit SecG	DB346_00895	DB345_13490	DB347_08140	DB354_11690
	secY	preprotein translocase secY subunit	DB346_13265	DB345_04370	DB347_05875	DB354_13935
	secA	preprotein translocase subunit SecA	DB346_17455, DB346_21070	DB345_14025	DB347_06030	DB354_14105
	ftsY	signal recognition particle-docking protein FtsY	DB346_23955	DB345_06485	DB347_03010	DB354_02010
		signal recognition particle protein	DB346_15875	DB345_18055	DB347_09795	DB354_10685
**Type IV pilus components and biogenesis**						
	rpoN	RNA polymerase sigma-54 factor RpoN	DB346_03420	DB345_18120	DB347_11420	DB354_15055
	pilA/flp	pilus assembly protein Flp/PilA	-	-	* 645	-
	pilB	Type IV fimbrial assembly, ATPase PilB	DB346_03220	DB345_16865	* 1762	* 3198, 3736
	pilC	Type IV fimbrial assembly protein PilC	DB346_03215	DB345_12000	* 1608	* 120, 1916, 1998
	pilD/pppA	leader peptidase (prepilin peptidase) / N-methyltransferase	* 3334	* 415	* 682	* 1649
	pilH	twitching motility protein PilH	* 4352	-	-	* 3144

pilS	sensor protein PilS	-	-	* 1270, 2704	* 198
pilQ	Type IV pilus biogenesis protein PilQ	* 1892	* 35, 523, 1685, 2306	* 1434	* 2102
pilT	Twitching motility protein PilT	DB346_00455, DB346_21030, DB346_21135	DB345_04040, DB345_04045, DB345_14265	DB347_18060, DB347_03210, DB347_03215	DB354_11370
pilJ	type IV pilus biogenesis protein PilJ	* 1941, 4922	-	* 4813	* 3146
pilM	Type IV pilus biogenesis protein PilM	* 2289	* 527	-	-
pilU	Twitching motility protein PilU	-	-	-	* 523
rcpA/cpaC	Type II/IV secretion system secretin RcpA/CpaC, associated with Flp pilus assembly	* 4020	* 522	-	-
rcpC/cpaB	Flp pilus assembly protein RcpC/CpaB	DB346_19230	-	-	-
tadB	Flp pilus assembly protein TadB	* 4024	-	-	-
tadC	Type II/IV secretion system protein TadC, associated with Flp pilus assembly	* 4025	-	-	-
cpaE/tadZ	Type II/IV secretion system ATPase TadZ/CpaE, associated with Flp pilus assembly	* 4021	-	-	-
cpaF/tadA	Type II/IV secretion system ATP hydrolase TadA/VirB11/CpaF, TadA subfamily	DB346_12315	-	-	-

**Flagellar Assembly/ Chemotaxis**					
cheA	two-component system, chemotaxis family, sensor kinase CheA	* 413, 5203	-	DB347_23285, DB347_23860, DB347_23905, DB347_11875	DB354_03550, DB354_21405
cheB	chemotaxis response regulator protein-glutamate methylesterase CheB	DB346_09420, DB346_24895	DB345_09260	DB347_15360	DB354_21385
cheD	chemotaxis protein CheD	DB346_10370	-	DB347_04350	DB354_19755, DB354_10605
cheR	chemotaxis protein methyltransferase CheR	DB346_10380	DB345_09245	DB347_07105, DB347_11895, DB347_23845, DB347_23890, DB347_04330	DB354_03565, DB354_21390, DB354_19770
cheW	positive regulator of CheA protein activity (CheW)	DB346_09445, DB346_24905, DB346_00145	DB345_09240	DB347_07120, DB347_08655, DB347_11885, DB347_23290, DB347_23850	DB354_03560
cheY	chemotaxis protein CheY	* 1973, 3396	-	* 4488, 2238, 4205, 4215	* 1118, 2956, 3689
cheX	chemotaxis protein CheX	DB346_09570, DB346_16380	-	DB347_16520	DB354_01045
mcp	methyl-accepting chemotaxis protein	DB346_20470	DB345_09235	DB347_15350, DB347_11890	* 224, 2666, 2958, 3032, +4
fliA	RNA polymerase sigma factor for flagellar operon FliA	DB346_02175	-	DB347_04210	DB354_19895

fliG	flagellar motor switch protein FliG	-	-	DB347_04305	DB354_19795
fliI	flagellum-specific ATP synthase	-	-	* 906	* 3365
fliP	flagellar biosynthetic protein FliP	-	-	* 895	DB354_19855
fliQ	flagellar biosynthetic protein FliQ	-	-	DB347_04240	DB354_19860
fliR	flagellar biosynthetic protein FliR	-	-	-	* 3377
flhA	flagellar biosynthesis protein FlhA	-	-	DB347_04225	DB354_19875
flhB	flagellar biosynthetic protein FlhB	DB346_06200	-	* 892	* 3378
fliL	flagellar basal body protein FliL	-	-	DB347_04265	DB354_19835
fliM	flagellar motor switch protein FliM	-	-	DB347_04260	DB354_19840
fliN/Y	flagellar motor switch protein FliN/Y	-	-	DB347_04255	DB354_19845
fliF	flagellar M-ring protein FliF	-	-	DB347_04310	DB354_19790
flgI	flagellar P-ring protein precursor FlgI	-	-	DB347_04470	DB354_19935
flgH	flagellar L-ring protein precursor FlgH	-	-	DB347_04465	DB354_19930
flgC	flagellar basal-body rod protein FlgC	-	-	DB347_04320	DB354_19780
flgG	flagellar basal-body rod protein FlgG	-	-	DB347_04455	DB354_19920

	flgM	flagellar biosynthesis protein FlgM	DB346_08350	-	-	-
	flgE	flagellar hook protein FlgE	-	-	DB347_09075	* 3370
	flgK	flagellar hook-associated protein 1 FlgK	-	-	DB347_04485	DB354_19950
	flgL	flagellar hook-associated protein 3 FlgL	-	-	* 949	DB354_19955
		flagellin	-	-	DB347_24395, DB347_24390, DB347_16470, DB347_16465	DB354_19690, DB354_19685, DB354_19970
**Adhesion**						
	aidA	Type V secretory pathway, adhesin AidA	-	* 1090, 1497, 2917	-	-
		putative adhesion protein	* 2516, 4002, 875, +6	-	* 287, 1252, 4051	* 1169, 1903
		putative hemagglutinin protein	* 319, 665	* 46, 1147	* 3507	-

Coding DNA sequences predicted with the NCBI Annotation Pipeline (PGAP)[1], KEGG/KAAS[2,3] or RAST[4,5] combined with affiliated gene locus tags. For each strain (*Verrucomicrobia* strains LW23, LR76, EW11 and ER46) gene names and the proposed function of gene products, putatively involved in interactions with plants is shown.

* gene(s) only detected by RAST/KEGG annotation
– gene(s) not detected.

[1] Tatusova T, Dicuccio M, Badretdin A, Chetvernin V, Nawrocki EP, Zaslavsky L *et al.*: NCBI prokaryotic genome annotation pipeline. *Nucleic Acids Res* 2016; **44**:6614–24.

[2] Kanehisa M, Furumichi M, Tanabe M, Sato Y, Morishima K: KEGG : new perspectives on genomes: pathways , diseases and drugs. *Nucleic Acids Res* 2017; **45**: 353–361.
[3] Moriya Y, Itoh M, Okuda S, Yoshizawa AC, Kanehisa M: KAAS : an automatic genome annotation and pathway reconstruction server. *Nucleic Acids Res* 2007; **35**: 182–185.
[4] Aziz RK, Bartels D, Best AA, Dejongh M, Disz T, Edwards RA *et al.* : The RAST Server : Rapid Annotations using Subsystems Technology. *BMC Genomics* 2008; **15**: 1–15.
[5] Overbeek R, Olson R, Pusch GD, Olsen GJ, Davis JJ, Disz T, *et al.:* The SEED and the Rapid Annotation of microbial genomes using Subsystems Technology (RAST). *Nucleic Acids Res* 2014; **42**: 206–214.

Supplementary Table S8. Assessment of enzymatic activities using APIzym[a]

Enzyme	*Strain*			
	strain LW23	strain LR 76	strain EW11	strain ER46
Alkaline phosphatase [b]	+	+	+	(+)
Esterase (C4)	+	+	+	(+)
Esterase lipase (C8)	(+)	+	-	(+)
Lipase (C14)	-	-	-	-
Leucine arylamidase	+	-	(+)	+
Valine arylamidase	+	-	(+)	+
Cysteine arylamidase	(+)	-	-	-
Trypsin	(+)	-	-	-
α-chymotrypsin	(+)	-	-	-
Acid phosphatase	+	+	+	+
Naphthol-AS-BI-phosphohydrolase	+	+	(+)	+
α-galactosidase	(+)	+	+	+
β-galactosidase	+	+	+	+
β-glucuronidase	-	-	+	-
α-glucosidase	-	-	-	-
β-glucosidase	-	-	(+)	(+)
N-acetyl-β-glucosaminidase	-	-	+	+
α-mannosidase	(+)	-	(+)	(+)
α-fucosidase	+	(+)	(+)	(+)

[a] +, positive result for bioassay; (+), weak positive result; -, negative result.

[b] Main enzyme activities were determined by using an API ZYM kit [1]. All strains were tested positive in varying strength for the reaction of esterase (C4), naphthol-AS-BI-phosphohydrolase, α- and β-galactosidase, α-fucosidase as well as alkaline and acid phosphatase. The strongest reaction on phosphatase activity showed strain EW11, indicating a possible role in phosphate solubilisation. Lipase (C14) and α-glucosidase were not detected in any strain. Strains LW23 and ER46 were tested positive for valine and leucine arylamidase, which was described to be active and abundant in soils, being involved in the release of amino acids from soil organic matter [2], likely promoting also nitrogen cycling. In contrast to the other strains, only strain LW23 showed a weak trypsin and α-chymotrypsin activity, which are proteolytic enzymes and may have a complex and inhibitory effect on bacterial growth by destroying bacterial cell wall structures [3,4].

1. Humble, M. W., King, A. & Phillips, I. Api Zym - Simple Rapid System for Detection of Bacterial Enzymes. *J Clin Pathol* **30**, 275-277 (1977).
2. Acosta-Martinez, V. & Tabatabai, M. A. Arylamidase activity in soils: effect of trace elements and relationships to soil properties and activities of amidohydrolases. *Soil Biol. Biochem.* **33**, 17-23 (2001).
3. Blow, D. M. Structure and Mechanism of Chymotrypsin. *Acc. Chem. Res.* **9**, 145-152 (1976).
4. Zhang, X. H. *et al.* A complex of trypsin and chymotrypsin effectively inhibited growth of pathogenic bacteria inducing cow mastitis and showed synergistic antibacterial activity with antibiotics. *Livest Sci* **188**, 25-36 (2016).
